# *FNDC3B* circular RNA promotes the migration and invasion of gastric cancer cells via the regulation of E‐cadherin and CD44 expression

**DOI:** 10.1002/jcp.28588

**Published:** 2019-04-08

**Authors:** Yuling Hong, Haifeng Qin, Yin Li, Yuhai Zhang, Xunrong Zhuang, Lei Liu, Kun Lu, Long Li, Xiaoling Deng, Fan Liu, Songlin Shi, Guoyan Liu

**Affiliations:** ^1^ Department of Gastrointestinal Surgery Zhongshan Hospital of Xiamen University Xiamen Fujian China; ^2^ Department of Basic Medicine Medical College of Xiamen University Xiamen Fujian China; ^3^ Department of Pulmonary Neoplasm Internal Medicine The 307th Hospital of Military Chinese People's Liberation Army Beijing China; ^4^ Department of Medical Examination Xiamen International Travel Healthcare Center Xiamen Fujian China; ^5^ Department of Cancer Prevention and Rehabilitation, Huayan Science and Technology Cancer Prevention and Rehabilitation Research Center School of Pharmaceutical Sciences Xiamen University Fujian China; ^6^ Department of Orthopedics The Second Affiliated Hospital of Fujian Medical University Fujian China

**Keywords:** *CD44*, circRNA, *FNDC3B*, IGF2BP3, migration

## Abstract

Circular RNAs (circRNAs) are a new class of RNAs, and many studies have identified thousands of circRNAs in tumor cells. Fibronectin type III domain‐containing protein 3B (*FNDC3B*) circular RNA (circFNDC3B, circBase ID: hsa_circ_0006156) circularizes with exons 5 and 6. Gibson Assembly DNA technology was used to construct a circFNDC3B expression vector without a splice site and restriction enzyme site. We showed that circFNDC3B increased migration and invasion in gastric cancer (GC). Ectopic expression of circFNDC3B reduced the level of E‐cadherin protein to promote the epithelial–mesenchymal transition in GC. RNA immunoprecipitation assays and RNA pull‐down assays confirmed that circFNC3B increased CD44 expression, which was associated with cell adhesion, via the formation of a ternary complex of circFNDC3B‐IGF2BP3‐*CD44* mRNA. These results indicated that circFNDC3B was associated with the degree of malignancy to highlight the specific characteristics of cell invasion.

## 1 INTRODUCTION

Gastric cancer (GC) is a malignancy with a high incidence and mortality, especially in developing countries (Hartgrink, Jansen, van Grieken, & van de Velde, 2009). GC symptoms are not really apparent at an early stage, which leads to poor prognosis. Recently, many oncogenes or tumor suppressors have been identified as key players in GC tumorigenesis. However, little is known about the role of these entities in the control mechanisms involved in GC. Therefore, exploration of new regulators and therapeutic targets is essential to understand the particular molecular mechanisms underlying GC development.

Circular RNAs (circRNAs) are recognized as endogenous noncoding RNAs that ubiquitously exist in eukaryotic cells. However, some studies have shown that some circRNAs can translate proteins (Pamudurti et al., 2017; Yang et al., 2017). CircRNAs are circularized by covalently joining the 3′ end to the 5′ end of the RNA molecule. This closed‐loop structure displays resistance to digestion with RNase R exonuclease (Vincent & Deutscher, 2006). Existing research indicates that circRNAs play an important role in regulating gene expression in eukaryotic cells (Ashwal‐Fluss et al., 2014; Z. Li et al., 2015; Zheng et al., 2016). A recent study profiled the differential expression of circRNAs in GC tissues, including 107 upregulated and 201 downregulated circRNAs and suggested that chr1, chr2, chr3, chr9, and chr17 are the most prominent chromosomes in GC (Shao et al., 2017). According to these data, circFNDC3B is produced by fibronectin type III domain‐containing protein 3B (*FNDC3B*) located on chr3. The deregulation of FNDC3B increases cell migration (Cai et al., 2012; Zhang et al., 2009). However, very little is known about the biological function of *FNDC3B* and circFNDC3B.

Here, our data demonstrated that circFNDC3B could influence cell migration and invasion in GC. Overexpression of circFNDC3B regulated the expression of E‐cadherin. Moreover, circFNDC3B regulated CD44 expression by a novel mechanism, forming a ternary complex of circFNDC3B‐IGF2BP3‐*CD44* mRNA in GC. Insulin‐like growth factor 2 binding protein 3 (IGF2BP3) is an RNA binding protein associated with a variety of malignant tumors and can bind to circFNDC3B (Ennajdaoui et al., 2016; Schneider et al., 2016). Moreover, IGF2BPs can target *CD44* mRNA and increase its stability (Stohr & Huttelmaier, 2012). Taken together, our data suggested that when circFNDC3B was upregulated, it promoted cell migration and invasion of GC cells via the formation of a ternary complex of circFNDC3B‐IGF2BP3‐*CD44* mRNA and the modulation of E‐cadherin in GC.

## 2 MATERIALS AND METHODS

### 2.1 Materials

The monoclonal or polyclonal antibodies against FNDC3B (catalog No. 22605‐1‐AP), IGF2BP3 (catalog No. 14642‐1‐AP), CD44 (catalog No. 15675‐1‐AP), and flag–tag (catalog No. 20543‐1‐AP) were purchased from Proteintech Group (Wuhan, China). Horseradish peroxidase (HRP)‐conjugated goat antimouse IgG and HRP‐conjugated goat antirabbit IgG were obtained from Pierce (Thermo Fisher Scientific, MA). RNA RT and polymerase chain reaction (PCR) kits were purchased from TaKaRa (Dalian, China). ChamQ SYBR qPCR Master Mix (Real‐time PCR kits) was obtained from Vazyme (Nanjing, China). Gibson Assembly DNA (HB‐infusion TM) kits were obtained from HANBIO (Shanghai, China). Lipofectamine‐3000 kits were obtained from Thermo Fisher Scientific. Protein A/G‐Sepharose beads were obtained from Millipore (Merck, NJ). Streptavidin MagBeads was obtained from GenScript (NJ).

### 2.2 Cell lines

Human GC cell lines AGS, SGC‐7901, MGC‐803, and BGC‐823, as well as gastric epithelium cell line GES‐1, were purchased from the China Center for Type Culture Collection and cultured in RPMI‐1640 medium (Gibco, Thermo Fisher Scientific) containing 10% fetal bovine serum (FBS; Gibco, Thermo Fisher Scientific). The cells were cultured at 37°C with 5% CO_2_.

### 2.3 Plasmid and small interfering RNA

A plasmid (pcDNA3.1 [+] CircRNA Mini Vector) containing the splice sites, along with short (~30–40 nt) inverted repeats of the ZKSCAN1 introns upstream and downstream of exons 2 and 3, was purchased from the nonprofit plasmid repository Addgene (#60648). This plasmid was selected to allow intervening exons to form a circular RNA in the cell. We used this construct to express human *FNDC3B* circular RNA (circFNDC3B).

To generate circFNDC3B without splice sites or restriction enzyme sites, we used Gibson Assembly DNA technology (HB‐infusion TM) and designed two pairs primers of the vector and fragment of FNDC3B exons 5 and 6 with the SnapGene 2.3.2 software (GSL Biotech LLC, Chicago). The vector and fragment were synthesized using PCR (TaKaRa).

According to the circFNDC3B junction sequence, we designed three small interfering RNAs (siRNAs) specifically targeting circFNDC3B, and DNA oligo probes labeled with biotin against the endogenous or ectopic expression of circFNDC3B were synthesized. In addition, three siRNAs specifically targeting IGF2BP3 were purchased from Sigma‐Aldrich (Merck KGaA, NJ).

### 2.4 Western blot analysis

Cells were harvested and lysed in radioimmunoprecipitation assay lysis buffer, and protein samples were separated by sodium dodecyl sulfate‐polyacrylamide gel electrophoresis and then transferred to polyvinylidene difluoride membranes. The membranes were blocked with 5% defatted milk for 1 hr at room temperature (RT) and then incubated with the primary antibody diluted in TBST overnight at 4°C. The membranes were washed with TBST buffer and incubated with secondary antibodies conjugated to HRP. The band signals were visualized by enhanced chemiluminescence (Pierce) and quantified with Image J 1.46r (NIH, MD).

### 2.5 Reverse transcription‐PCR and real‐time PCR

In brief, cells were harvested and total RNA was extracted. One microgram of total RNA was used to synthesize cDNA. Real‐time PCR was performed with ChamQ SYBR qPCR Master mix using 2 μl of cDNA as templates. The primers used for real‐time PCR and human 18S rRNA served as internal controls.

### 2.6 Wound‐healing assay

Cells were seeded in six‐well plates and grown to 70 to 80% confluence. Then, cells were transfected with plasmid or siRNA using Lipofectamine‐3000 according to the manufacturer's instructions. After 48 hr, when the cells reached 90% confluence, a wound gap was carefully scratched on the plate of cells with a sterile 10 μl pipette tip. The scraped cells were removed with PBS. Then, the cells were again cultured in serum‐free medium. The wound‐healing process, during which cells moved into the wound gap, was recorded by microscope, and images were taken at random wound areas using an inverted microscope (Olympus IX51, Japan).

### 2.7 Transwell invasion assay

According to the manufacturer's instructions, the invasion assay was performed using Transwell chambers (8 μm pore size; Corning, NY). The cells were transfected with plasmid or siRNA as described above. After 48 hr, the cells were trypsinized and resuspended in serum‐free RPMI‐1640 medium. A total of 2 × 10^5^ MGC‐803 or 3 × 10^5^ BGC‐823 cells/well in 200 μl serum‐free medium were plated in the upper chamber with a coated extracellular matrix (ECM) gel (BD Bioscience, Franklin Lakes, NJ) for the invasion assay. RPMI‐1640 medium with 10% FBS was added into the lower chamber. After incubation for 24 hr, cells that did not invade were mechanically removed with a cotton swab, and the membranes were fixed with 4% paraformalin for 20 min. Then, the cells were stained with crystal violet for 30 min at RT. Three different areas of each well were randomly selected for imaging by microscopy (Olympus IX51). Cell numbers were quantified by Image Pro Plus 6.0 (Media Cybernetics, MD).

### 2.8 RNA immunoprecipitation assay

RNA immunoprecipitation (RIP) assays were performed as previously described (Dahm et al., 2012). First, appropriate A/G‐Sepharose slurry protein was prepared with NT2 buffer (consisting of Tris‐HCl [pH 7.4], NaCl, MgCl_2_, NP40) containing 5% bovine serum albumin and incubated at 4°C overnight with rotation. Cells were transfected with plasmid or siRNA as described above. After 48 hr, the cells were washed three times with ice‐cold PBS and then lysed in 1 ml of (PLB) Polysome lysis buffer, (consisting of KCl, MgCl_2_, HEPES [pH 7.0], NP40, DTT (DL‐Dithiothreitol)) containing RNase inhibitor (TaKaRa) and protease inhibitor (Roche, Basel, Switzerland) on ice for 30 min. The protein A/G‐Sepharose was washed five times with NT2 buffer containing RNase inhibitor and resuspended in 700 μl ice‐cold NT2 buffer. The slurry was equally distributed to each cell lysate, and the corresponding primary antibody or IgG was added and incubated while rotating at 4°C overnight. Pellets were washed five times with NT2 buffer containing RNase inhibitor. The eluted coprecipitated RNA was analyzed by quantitative reverse transcription‐PCR (qRT‐PCR) to determine the binding products using corresponding primers.

### 2.9 RNA pull‐down assay

The RNA pull‐down assay was performed as previously described (Bai, Bai, & Sun, 2016). Briefly, according to the manufacturer's instructions (GenScript), streptavidin MagBeads were diluted with binding buffer to bind to biotin‐labeled oligonucleotides at 4°C overnight with rotation. The cells were transfected with plasmid or siRNA as described previously. After 48 hr, the cells were washed three times with ice‐cold PBS and lysed in 1 ml of hypotonic buffer (consisting of Tris‐HCl [pH 7.4], KCl, MgCl_2_, DTT, PMSF [Phenylmethanesulfonyl fluoride]) containing RNase inhibitor and protease inhibitor on ice for 30 min. Then, the cell lysate was treated with either scrambled oligonucleotide or circFNDC3B oligonucleotide for 3 hr at 4°C with rotation. The pellets were washed five times with binding buffer containing RNase inhibitor and a protease inhibitor. The pull‐down of RNA or protein was subjected to qRT‐PCR analysis or western blot to determine the binding products.

### 2.10 Statistical analysis

All of the experiments were performed in triplicate. The GraphPad 6.0 software (Graphpad, San Diego, CA) was used to analyze the data. All the data were subject to an independent sample *t* test and expressed as the mean ± standard error of the mean. The levels of significance were set at *p < 0.05, **p < 0.01, and ***p < 0.0001.

## 3 RESULTS

### 3.1 Expression of circFNDC3B in GC cells

**Figure 1:**
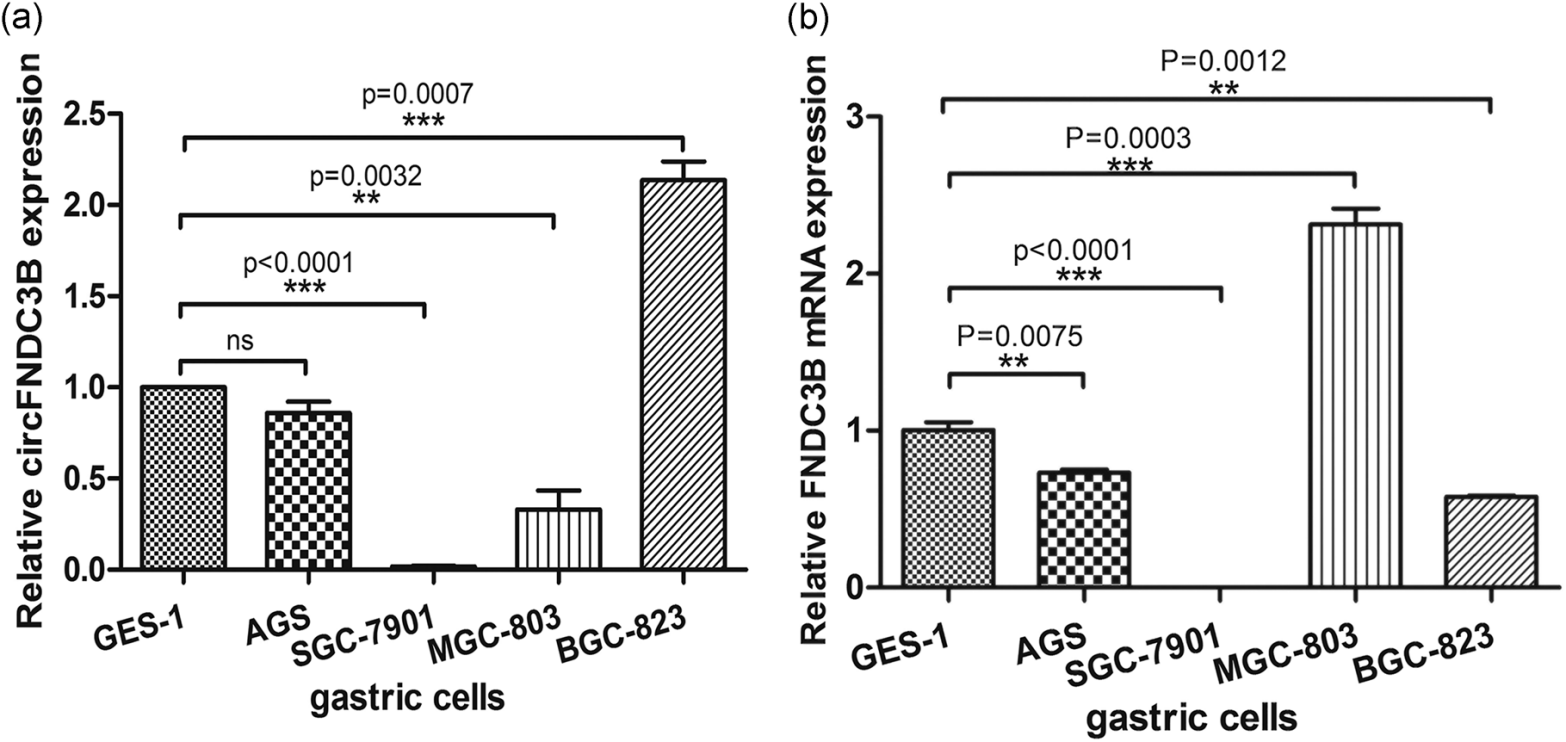
Expression of circFNDC3B in gastric cancer cells. (a) The level of circFNDC3B was analyzed by qRT‐PCR in control cells from the gastric epithelium cell line GES‐1, as well as in the AGS, SGC‐7901, BCG‐823 and MGC‐803 gastric cancer cell lines. (b) The expression level of *FNDC3B* mRNA was analyzed by qRT‐PCR in control cells from the gastric epithelium cell line GES‐1, as well as in the AGS, SGC‐7901, BCG‐823, and MGC‐803 gastric cancer cell lines. Data were expressed as the mean ± *SEM* and were analyzed by independent samples *t* test, *n* = 3. *p < 0.05, **p < 0.01, ***p < 0.0001. FNDC3B: fibronectin type III domain‐containing protein 3B; qRT‐PCR: quantitative reverse transcription‐PCR; *SEM*: standard error of the mean

First, according to the circBase database and the CircInteractome tool (Dudekula et al., 2016; Glazar, Papavasileiou, & Rajewsky, 2014), we designed a divergent primer based on circRNA junction sequences and specifically targeted circFNDC3B to accurately detect circFNDC3B levels by RT‐qPCR analysis (Du et al., 2016; Z. Li et al., 2015). To test whether circFNDC3B played an important role in gastric development, we examined circFNDC3B expression in five gastric cell lines by qRT‐PCR. We found that the level of circFNDC3B was very different between cell lines (Figure 1a). The AGS cancer cell line expressed the same amount of circFNDCB3 as the control gastric epithelium cell line GES‐1 and SGC‐7901 cell did not express circFNDC3B at all, and MGC‐803 expressed a lower amount than GES‐1, whereas BCG‐823 cells expressed a much higher amount of circRNA than the GES‐1 control cells. Moreover, the expression levels of *FNDC3B* mRNA were detected in these five cells, and the level of *FNDC3B* mRNA was also different between cell lines (Figure 1b).

The GC cells that expressed the highest amount of circFNDC3B, BGC‐823 cells, also expressed the most poorly differentiated phenotype with high migration and invasion rates, suggesting that the level of circFNDC3B expression is related to the degree of differentiation of GC cells (Bloushtain‐Qimron et al., 2008; Q. F. Li, Ou‐Yang, Li, & Hong, 2000; YuHui Cai, Zhang, Lu, Zhou, 1986). Therefore, we investigated whether circFNDC3B controls FNDC3B expression.

### 3.2 CircFNDC3B did not influence FNDC3B expression

**Figure 2:**
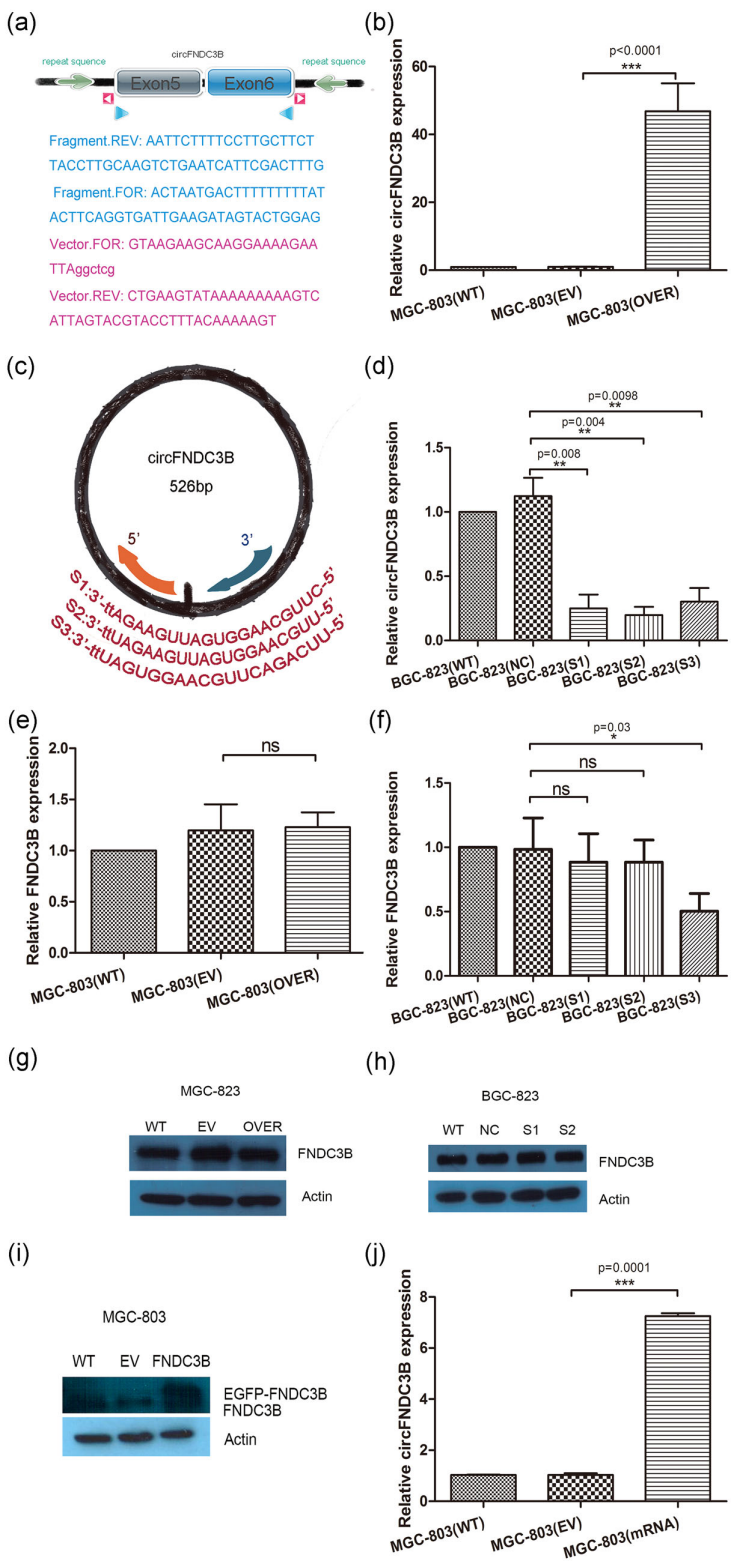
CircFNDC3B did not influence FNDC3B expression. (a) The pcDNA3.1‐circFNDC3B‐mini vector was constructed without a restriction enzyme site by Gibson Assembly DNA clone technology. The light green arrows show the inverted repeat supporting circFNDC3B circularization. Pink triangle primers or blue triangle primers were designed to amplify vector or circFNDC3B exons 5 and 6. (b) The circFNDC3B expression vector successfully induced an increase in circFNDC3B expression in MGC‐803 cells (EV: empty vector; OVER: overexpressing circFNDC3B; WT: wild‐type). (c) The three siRNAs targeting the circFNDC3B junction sequence were designed and only exhibited the sense strand. (d) BGC‐823 cells were transfected with three siRNAs specifically targeting circFNDC3B (S1, S2, S3) or a negative control siRNA for 48 hr, and the level of silencing circFNDC3B was detected by qRT‐PCR (WT: wild‐type; NC: negative control; siRNA: S1, S2, S3). (e,g) MGC‐803 cells were transfected with the circFNDC3B vector or an empty vector. Cells were cultured for 48 hr, and then we examined *FNDC3B* mRNA and protein expression by qRT‐PCR and western blot. The results showed that overexpression of circFNDC3B did not significantly increase the mRNA or protein level of *FNDC3B* (WT: wild‐type; EV: empty vector; OVER: overexpressing circFNDC3B). (f,h) BGC‐823 cells were transfected with siRNA (S1, S2, S3) or a negative control siRNA. Cells were cultured for 48 hr, and then we examined circFNDC3B and *FNDC3B* mRNA and protein expression by qRT‐PCR and western blot, respectively. The silencing of circFNDC3B with S1 and S2 did not statistically affect *FNDC3B* mRNA or protein levels (WT: wild‐type; NC: negative control; siRNA: S1, S2, S3). (i,j) MGC‐803 cells were transfected with the pEGFP‐FNDC3B vector or an empty vector. After 48 hr, FNDC3B levels and circFNDC3B levels were detected by western blot and qRT‐PCR. The results suggest that *FNDC3B* mRNA increased circFNDC3B (WT: wild‐type; EV: empty vector against EGFP‐FNDC3B; *FNDC3B* mRNA: overexpressing *FNDC3B* mRNA). Data were expressed as the mean ± *SEM* and were analyzed by independent samples *t* test, *n* = 3. *p < 0.05, ^**^ *p* < 0.01, ^***^ *p* < 0.0001. FNDC3B: fibronectin type III domain‐containing protein 3B; qRT‐PCR: quantitative reverse transcription‐PCR; *SEM*: standard error of the mean; siRNA: small interfering RNA [Color figure can be viewed at wileyonlinelibrary.com]

Based on the expression of circFNDC3B in GC cells, we chose MGC‐803 cells with low expression of circFNDC3B and BGC‐823 cells with high expression of circFNDC3B to interfere with the level of circFNDC3B. We structured the circFNDC3B expression vector and designed siRNAs to explore the role of circFNDC3B. A construct containing circFNDC3B transcripts without a restriction enzyme site by the Gibson Assembly DNA clone technology was transfected into MGC‐803 cells and compared with an empty vector transfection for 48 hr (Figure 2a). qRT‐PCR and Sanger sequencing were performed to validate the resistance of circFNDC3B to RNase R digestion in MGC‐803 (Figure S1). The efficiency of circFNDC3B overexpression was subsequently confirmed by qRT‐PCR analysis. The results suggested that the circFNDC3B expression vector successfully increased circFNDC3B expression in MGC‐803 cells (Figure 2b). Similarly, we designed three siRNAs specifically targeting endogenous circFNDC3B to silence circFNDC3B (Figure 2c). BGC‐823 cells, which highly expressed circFNDC3B, were transfected with three siRNAs specifically targeting circFNDC3B or a negative control siRNA for 48 hr. According to the qRT‐PCR results, we found that all three siRNAs could reduce circFNDC3B, but S3 also influenced *FNDC3B* mRNA levels (Figure 2d,f). S3 silenced 59.44% of *FNDC3B* mRNA; therefore, we selected S1 and S2 to silence circFNDC3B.

Studies have shown that the homozygous disruption of *FNDC3B* has a lethal effect on the migration of mouse embryo fibroblasts and that FNDC3B participates in several cancer pathways (Cai et al., 2012; Nishizuka et al., 2009). In addition, deregulation of FNDC3B promotes cell migration (Zhang et al., 2009). Therefore, we needed to explore the relationship between *FNDC3B* mRNA and circFNDC3B. The results showed that the overexpression of circFNDC3B did not affect the level of *FNDC3B* mRNA or protein (Figure 2e,g). In addition, the silencing of circFNDC3B with S1 and S2 did not affect the level of *FNDC3B* mRNA or protein (Figure 2f,h). In addition, we constructed EGFP‐FNDC3B to explore whether FNDC3B influenced circFNDC3B and found that *FNDC3B* mRNA increased circFNDC3B levels (Figure 2i,j). However, based on this result, we did not yet determine the differences in the expression levels of circFNDC3B and *FNDC3B* mRNA, which might be involved in the mechanism of the upstream regulation.

### 3.3 CircFNDC3B promoted migration and invasion

**Figure 3:**
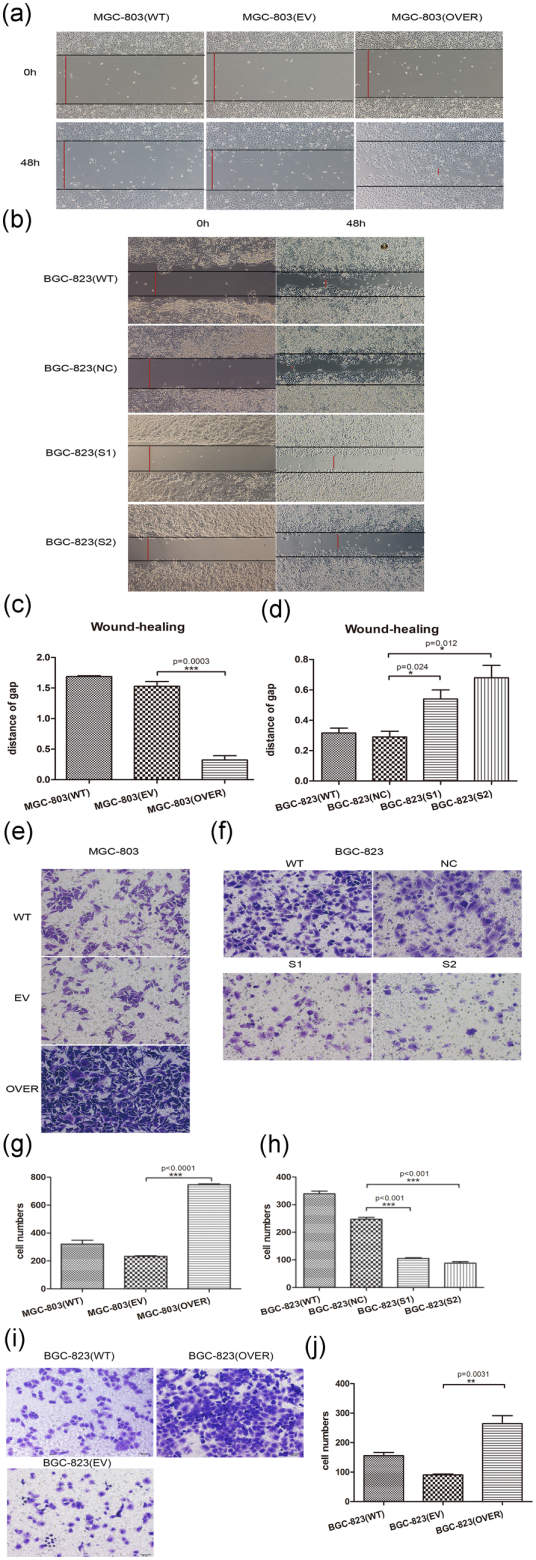
CircFNDC3B promoted migration and invasion. (a,b) A wound‐healing assay was performed to evaluate the migration ability of MGC‐803 and BGC‐823. MGC‐803 cells were transfected with circFNDC3B vector (OVER) or empty vector (EV) for 48 hr. BGC‐823 cells were transfected with siRNA (S1, S2) or negative control siRNA (NC) for 48 hr. A line was drawn from one end of the well to the other. The wound‐healing process was recorded by microscopy at 100× magnification. The red line indicates the measured distance after cell migration (WT: wild‐type; EV: empty vector; OVER: overexpressing circFNDC3B; NC: negative control; siRNA: S1, S2). (c,d) The migration ability was evaluated by the red line distance of the wound‐healing gap. Quantitative results showed that the wound gap was obviously shortened in MGC‐803 cells transfected with the circFNDC3B vector and that silencing circFNDC3B suppressed cell migration in BGC‐823 cells (WT: wild‐type; EV: empty vector; OVER: overexpressing circFNDC3B; NC: negative control; siRNA: S1, S2). (e,f) Transwell assays were performed to evaluate the invasion ability of MGC‐803 and BGC‐823 cells after overexpression and silencing of circFNDC3B. MGC‐803 cells were transfected with circFNDC3B vector (OVER) or empty vector (EV) for 48 hr. BGC‐823 cells were transfected with siRNA (S1, S2) or negative control siRNA (NC) for 48 hr. The cells were trypsinized, resuspended, and seeded on the upper chamber of well‐coated ECM gel. After 24 hr, the invaded cells on the bottom of the well were fixed and stained. The invaded cells stained by crystal violet were calculated as the degree of invasion. Three different areas of each well were randomly selected and observed under a microscope at 200× magnification. The results showed that MGC‐803 cells transfected with the circFNDC3B vector invaded more than the empty vector cells, whereas silencing circFNDC3B in BGC‐823 cells caused them to invade less than BGC‐823 cells with negative control (WT: wild‐type; EV: empty vector; OVER: overexpressing circFNDC3B; NC: negative control; siRNA: S1, S2). (g,h) Quantitative results showed that overexpressing circFNDC3B promoted cell invasion in MGC‐803 cells and that silencing circFNDC3B suppressed cell invasion in BGC‐823 cells (WT: wild‐type; EV: empty vector; OVER: overexpressing circFNDC3B; NC: negative control; siRNA: S1, S2). (i,j) BGC‐823 cells were transfected with the circFNDC3B vector, and results showed that circFNDC3B indeed promoted cell migration and invasion (WT: wild‐type; EV: empty vector; OVER: overexpressing circFNDC3B). Finally, we validated that circFNDC3B could promote cell migration and invasion. Data were expressed as the mean ± *SEM* and were analyzed by independent samples *t* test, *n* = 3. *p < 0.05, ^**^ *p* < 0.01, ^***^ *p* < 0.0001. ECM: extracellular matrix; FNDC3B: fibronectin type III domain‐containing protein 3B; *SEM*: standard error of the mean; siRNA: small interfering RNA [Color figure can be viewed at wileyonlinelibrary.com]

It is important to elucidate the mechanisms underlying circFNDC3B expression. We focused on cell migration and invasion, which were used to determine the degree of cancer malignancy. MGC‐803 cells transfected with the circFNDC3B vector were compared with those transfected with an empty vector for 48 hr. The wound‐healing assay and quantitative results showed that the wound gap was obviously shortened in MGC‐803 cells transfected with the circFNDC3B vector (Figure 3a,c). Similarly, a transwell assay was performed to assess the function of circFNDC3B in cell invasion. Wells were covered with ECM gel to evaluate cellular invasion ability. Consistently, the transwell assay and quantitative results indicated that ectopic expression of circFNDC3B promoted cell invasion in MGC‐803 cells (Figure 3e,g).

In addition, the silencing of circFNDC3B was detected in BGC‐823 cells transfected with S1 and S2 (Figure 3b,f). Quantitative results showed that silencing circFNDC3B suppressed cell migration and invasion in BGC‐823 cells (Figure 3d,h). In addition, BGC‐823 cells were transfected with the circFNDC3B vector, and the results showed that circFNDC3B indeed promoted cell migration and invasion (Figure 3i,j). Together, these results suggested that circFNDC3B promoted cell migration and invasion.

### 3.4 CircFNEC3B reduced the expression of E‐cadherin

**Figure 4:**
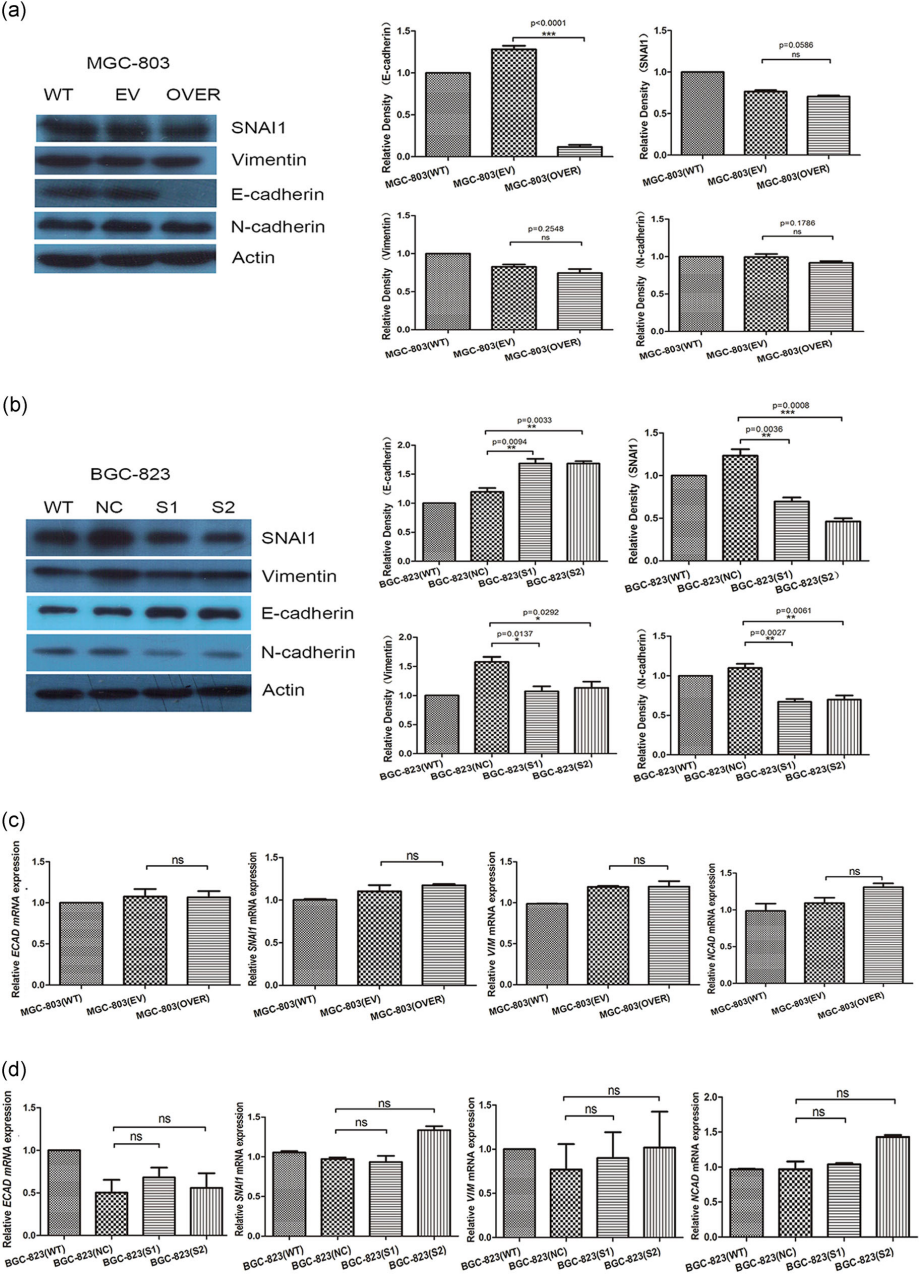
CircFNEC3B reduced the expression of E‐cadherin. (a) MGC‐803 cells were transfected with circFNDC3B vector (OVER) or empty vector (EV) for 48 hr. Overexpression of circFNDC3B only inhibited the expression of E‐cadherin protein. (WT: wild‐type; EV: empty vector; OVER: overexpressing circFNDC3B). (b) BGC‐823 cells were transfected with siRNA (S1, S2) or negative control siRNA (NC) for 48 hr. Silencing of circFNDC3B upregulated the expression of E‐cadherin and inhibited the expression of N‐cadherin, Vimentin, and SNAI1 in protein. (WT: wild‐type; NC: negative control; siRNA: S1, S2) (c,d) MGC‐803 cells were transfected with circFNDC3B vector (OVER) or empty vector (EV) for 48 hr. BGC‐823 cells were transfected with siRNA (S1, S2) or negative control siRNA (NC) for 48 hr. qRT‐PCR verified that circFNDC3B did not affect the expression of *ECAD (CDH1)* mRNA, *SNAI1* mRNA, *VIM* mRNA, and *NCAD (CDH2)* mRNA. (WT: wild‐type; EV: empty vector; OVER: overexpressing circFNDC3B; NC: negative control; siRNA: S1, S2). Data were expressed as the mean ± *SEM* and were analyzed by independent samples *t* test, *n* = 3. *p < 0.05, **p < 0.01, ***p < 0.0001. FNDC3B: fibronectin type III domain‐containing protein 3B; qRT‐PCR: quantitative reverse transcription‐PCR; *SEM*: standard error of the mean; siRNA: small interfering RNA [Color figure can be viewed at wileyonlinelibrary.com]

Cell migration and invasion are associated with the epithelial–mesenchymal transition (EMT). EMT is a process of transformation that drives epithelial‐derived tumor cells into malignant tumors (Cano et al., 2000). EMT induces epithelial tumor cells to migrate from the primary site to distant sites, which can cause distal metastasis of the tumor (Nieto, Huang, Jackson, & Thiery, 2016). Subsequently, to understand the mechanism underlying the robust effect of circFNDC3B in the invasion and migration of GC cells, mechanistic research was completed. By confirming the change in the marker protein during EMT, the downregulation of epidermal phenotype protein E‐cadherin can promote EMT (Thiery & Sleeman, 2006); the upregulation of mesenchymal phenotype protein N‐cadherin, Vimentin, and SNAI1 can promote EMT (Jolly et al., 2015; Thiery, Acloque, Huang, & Nieto, 2009). The ectopic expression of circFNDC3B inhibited the expression of E‐cadherin protein but did not affect the expression of N‐cadherin, Vimentin, or SNAI1 (Figure 4a). The silencing of circFNDC3B not only increased E‐cadherin levels but also reduced N‐cadherin, SNAI1 and Vimentin levels (Figure 4b). Moreover, circFNDC3B failed to regulate the levels of *ECAD (CDH1)* mRNA, *SNAI1* mRNA, *VIM* mRNA, and *NCAD (CDH2)* mRNA (Figure 4c,d). These results suggested that circFNDC3B promoted cell migration and invasion mainly by inhibiting the expression of E‐cadherin protein. BGC‐823 cells were transfected with the circFNDC3B vector, and the results showed that circFNDC3B indeed reduced the E‐cadherin level (Figure S1).

### 3.5 CircFNDC3B interacted with IGF2BP3 and *CD44* mRNA

**Figure 5:**
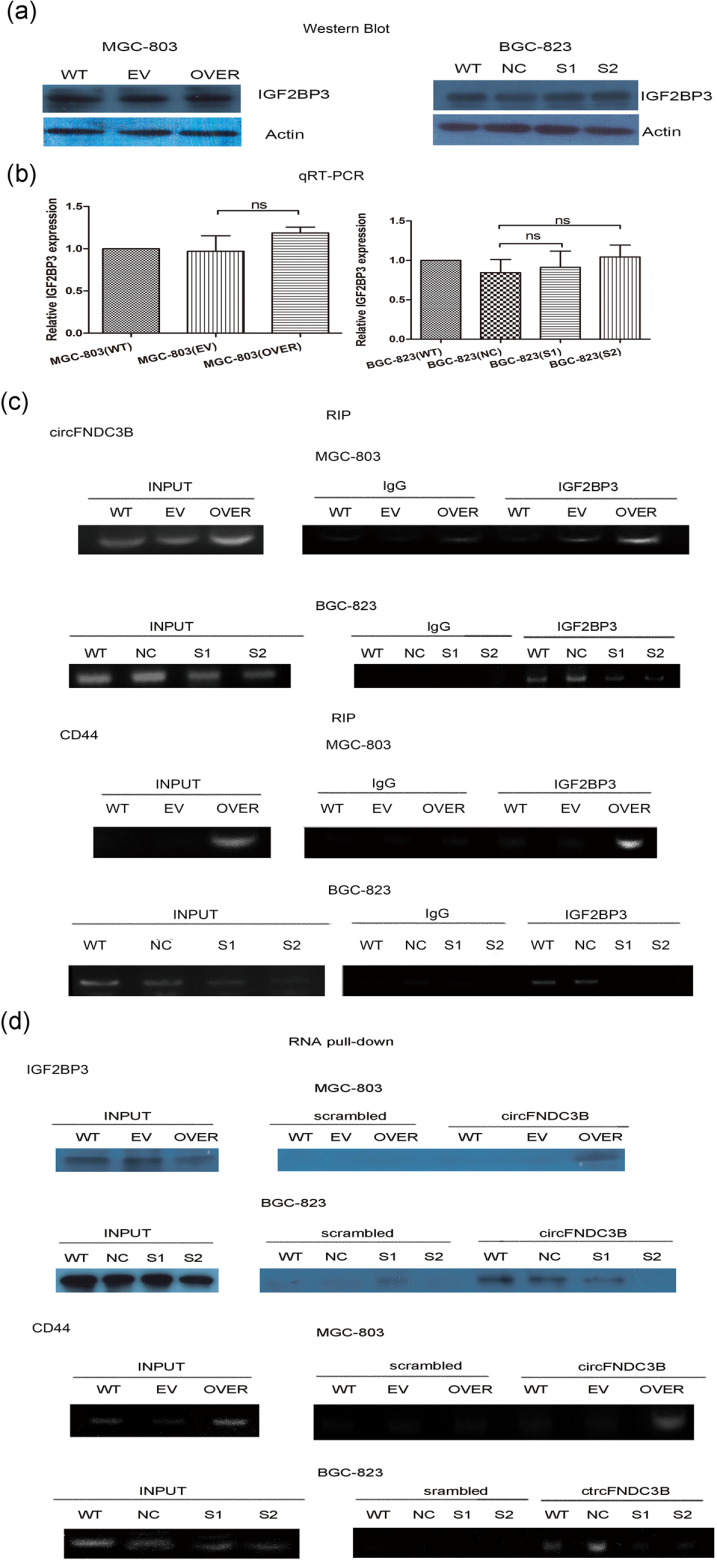
CircFNDC3B interacted with IGF2BP3 and CD44 mRNA. (a) The expression level of IGF2BP3 was evaluated in MGC‐803 and BGC‐823 cells by western blot. MGC‐803 cells were transfected with circFNDC3B vector (OVER) or empty vector (EV) for 48 hr. BGC‐823 cells were transfected with siRNA (S1, S2) or negative control siRNA (NC) for 48 hr. The results suggested that circFNDC3B did not significantly influence IGF2BP3 (WT: wild‐type; EV: empty vector; OVER: overexpressing circFNDC3B; NC: negative control; siRNA: S1, S2). (b) Ectopic expression or silencing of circFNDC3B did not statistically influence *IGF2BP3* mRNA expression by qRT‐PCR (WT: wild‐type; EV: empty vector; OVER: overexpressing circFNDC3B; NC: negative control; siRNA: S1, S2). (c) RIP assay using an antibody against IGF2BP3 was performed to evaluate the interaction between IGF2BP3, circFNDC3B, and *CD44* mRNA in MGC‐803 and BGC‐823 cell lines. CircFNDC3B or *CD44* mRNA was amplified by cirdFNDC3B primers or CD44 primers. The results showed that IGF2BP3 could interact with circFNDC3B and *CD44* mRNA by RT‐PCR (INPUT: total RNA was reverse‐transcribed with circFNDC3B primers or CD44 mRNA primers. IgG and IGF2BP3: after incubation with IgG or IGF3BP3 antibody). (d) RNA pull‐down was performed to assess the role of circFNDC3B between IGF2BP3 and *CD44* mRNA by the circFNDC3B biotinylated probe. Western blot detected the interaction between circFNDC3B and IGF2BP3. RT‐PCR assessed the interaction between circFNDC3B and *CD44* mRNA (INPUT: total protein or total RNA were incubated with IGF2BP3 antibody or reverse‐transcribed and amplified CD44 primers, scrambled and circFNDC3B: after incubation with biotin‐labeled scrambled oligonucleotide or circFNDC3B oligonucleotide). These results suggested that circFNDC3B could interact with IGF2BP3 and *CD44* mRNA. Finally, RIP and RNA pull‐down assays validated the ternary complex of circFNDC3B‐IGF2BP3‐*CD44* mRNA. (WT: wild‐type; EV: empty vector; OVER: overexpressing circFNDC3B; NC: negative control; siRNA: S1, S2). FNDC3B: fibronectin type III domain‐containing protein 3B; RIP: RNA immunoprecipitation; RT‐PCR: reverse transcription‐PCR; siRNA: small interfering RNA [Color figure can be viewed at wileyonlinelibrary.com]

Cell migration and invasion are also dynamic processes that depend on cell adhesion and cell motility. Because IGF2BP3, which regulates cell migration, could bind to circFNDC3B (Ennajdaoui et al., 2016; Schneider et al., 2016), we sought to detect IGF2BP3 levels in GC cells. We found that ectopic expression or silencing of circFNDC3B did not influence *IGF2BP3* protein and mRNA expression (Figure 5a,b). Next, we determined whether IGF2BP3 interacted with circFNDC3B by RIP assay. MGC‐803 cells and BGC‐823 cells were subjected to the RIP assay with an antibody against IGF2BP3, and the remaining RNAs were amplified by PCR with primers specific for circFNDC3B or *FNDC3B* mRNA. Our results showed that circFNDC3B was pulled down by antibody against IGF2BP3 (Figure 5c), whereas *FNDC3B* mRNA was not pulled down (Figure S1).

CD44 was correlated with cell migration and invasion (Polyak & Weinberg, 2009). IGF2BPs targeted *CD44* mRNA and increased its stability (Stohr & Huttelmaier, 2012; Vikesaa et al., 2006). To determine the molecular mechanism of circFNDC3B induction of cell migration and invasion, we analyzed the relationship between circFNDC3B, IGF2BP3, and *CD44* mRNA. The RIP assay confirmed that IGF2BP3 combined with *CD44* mRNA using primers specific for *CD44* mRNA (Figure 5c). Next, we performed an RNA pull‐down assay to validate the interaction between circFNDC3B and *CD44* mRNA and IGF2BP3. MGC‐803 cells and BGC‐823 cells were subjected to RNA pull‐down assays using a biotinylated probe against circFNDC3B. After pulling down with the probe, some of the lysates were used for western blotting with the IGF2BP3 antibody, and other lysates were subjected to PCR analysis for *CD44* mRNA. IGF2BP3 and *CD44* mRNA were successfully pulled down by the circFNDC3B probe (Figure 5d), whereas *FNDC3B* mRNA was not pulled down (Figure S1).

Both the RIP assay and RNA pull‐down assay showed that IGF2BP3 and *CD44* mRNA were increased after overexpression of circFNDC3B in MGC‐803 cells and conversely decreased with silencing of circFNDC3B in BGC‐823 cells. In summary, we concluded that circFNDC3B could interact with IGF2BP3 and *CD44* mRNA.

### 3.6 CircFNDC3B increased CD44 levels by binding to IGF2BP3

**Figure 6:**
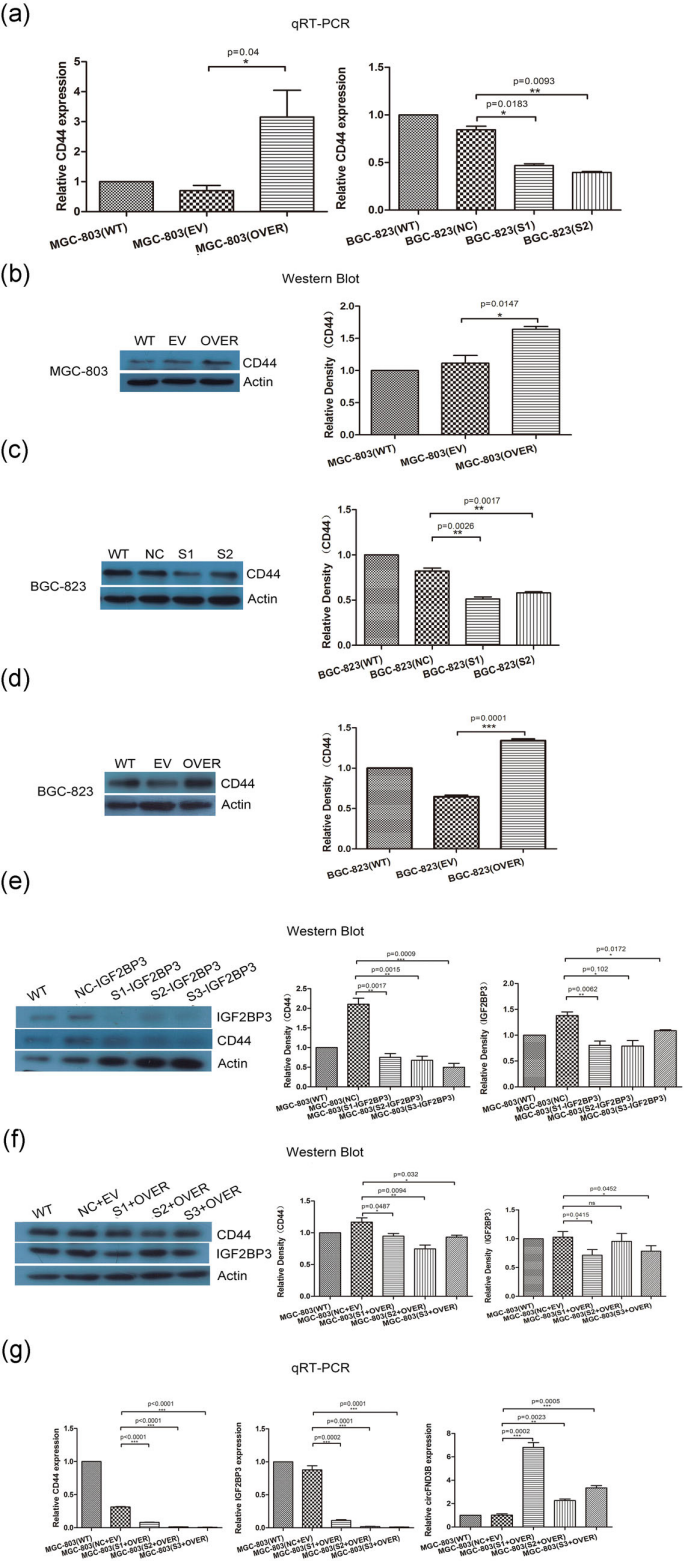
CircFNDC3B increased CD44 levels by binding to IGF2BP3. (a) The level of *CD44* mRNA in MGC‐803 and BGC‐823 cells was evaluated by qRT‐PCR. MGC‐803 cells were transfected with circFNDC3B vector (OVER) or empty vector (EV) for 48 hr. BGC‐823 cells were transfected with siRNA (S1, S2) or negative control siRNA (NC) for 48 hr. The results suggested that ectopic expression of circFNDC3B increased *CD44* mRNA, whereas silencing of circFNDC3B reduced *CD44* mRNA (WT: wild‐type; EV: empty vector; OVER: overexpressing circFNDC3B; NC: negative control; siRNA: S1, S2). (b,c) Western blot was performed to evaluate CD44 protein in MGC‐803 and BGC‐823 cells. The gray scale quantitative results suggested ectopic expression of circFNDC3B remarkably upregulated CD44 levels, and inversely, silencing of circFNDC3B downregulated CD44 levels. (d) We also overexpressed circFNDC3B on BGC‐823 cells, and the results showed that overexpression of circFNDC3B increased CD44 expression (WT: wild‐type; EV: empty vector; OVER: overexpressing circFNDC3B). (e) We detected the role of IGF2BP3 between circFNDC3B and *CD44* mRNA. MGC‐803 cells were transfected with siRNA against IGF2BP3 (S1, S2, S3) to detect the level of silencing IGF2BP3 and CD44 expression by Western blot. The results showed that silencing IGF2BP3 obviously reduced CD44 expression (WT: wild‐type; NC‐IGF2BP3: negative control; S1‐IGF2BP3, S2‐IGF2BP3, S3‐IGF2BP3: siRNA targeting IGF2BP3). (f) Then, MGC‐803 cells were transfected with siRNA against IGF2BP3 and transfected with the circFNDC3B vector again after 48 hr. Western blotting was performed to detect CD44 levels. The gray scale quantitative results suggested that when IGF2BP3 was silenced in MGC‐803 cells, overexpression of circFNDC3B did not truly increase the expression of CD44 (WT: wild‐type; NC+EV: negative control siRNA and empty vector; S1+OVER, S2+OVER, S3+OVER: siRNA against IGF2BP3 and circFNDC3B vector). (g) qRT‐PCR results showed that *IGF2BP3* mRNA and *CD44* mRNA and circFNDC3B levels were regulated (WT: wild‐type; NC+EV: negative control siRNA and empty vector; S1+OVER, S2+OVER, S3+OVER: siRNA against IGF2BP3 and circFNDC3B vector). Data were expressed as the mean ± *SEM* and were analyzed by independent samples *t* test, *n* = 3. *p < 0.05, ^**^ *p* < 0.01, ^***^ *p* < 0.0001. FNDC3B: fibronectin type III domain‐containing protein 3B; qRT‐PCR: quantitative reverse transcription‐PCR; *SEM*: standard error of the mean; siRNA: small interfering RNA [Color figure can be viewed at wileyonlinelibrary.com]

To further assess the mechanism of the circFNDC3B‐IGF2BP3‐*CD44* mRNA ternary complex, qRT‐PCR analysis and western blot assay were performed to examine the expression of *CD44* mRNA and protein. The results suggested that ectopic expression of circFNDC3B increased *CD44* mRNA, whereas silencing of circFNDC3B reduced *CD44* mRNA, as shown by qRT‐PCR assay (Figure 6a). Consistent with this result, the western blot assay suggested that ectopic expression of circFNDC3B increased CD44 levels by 35%, whereas silence of circFNDC3B reduced CD44 levels by 30% (Figure 6b,c). In addition, BGC‐823 cells were transfected with the circFNDC3B vector, and the results showed that overexpression of circFNDC3B increased CD44 expression (Figure 6d). Together, we, therefore, inferred that circFNDC3B could increase CD44 levels. Next, we also explored how IGF2BP3 affected the relationship between circFNDC3B and *CD44* mRNA. We disrupted the ternary complex by silencing IGF2BP3 in MGC‐803 cells and validated that circFNDC3B regulated the expression of CD44 by IGF2BP3. First, we designed three siRNAs against IGF2BP3 and detected the level of IGF2BP3 by western blot. The results showed that silencing IGF2BP3 obviously reduced CD44 expression (Figure 6e). Second, we disrupted the ternary complex by silencing IGF2BP3and then transfected the circFNDC3B vector into MGC‐803 cells. From the western blot and qRT‐PCR data, when IGF2BP3 was silenced in MGC‐803 cells, overexpression of circFNDC3B did not increase the expression of CD44 very well (Figure 6f,g). Based on these results, we inferred that circFNDC3B promoted CD44 expression via IGF2BP3 and that IGF2BP3 could affect the function of circFNDC3B to a certain extent.

Overall, we believed that the role of circFNDC3B was a mediator between IGF2BP3 and *CD44* mRNA via the formation of a ternary complex, which then promoted IGF2BP3 to promote CD44 levels, eventually leading to cell migration and invasion in GC.

### 3.7 CircFNDC3B had protein‐coding activity

**Figure 7:**
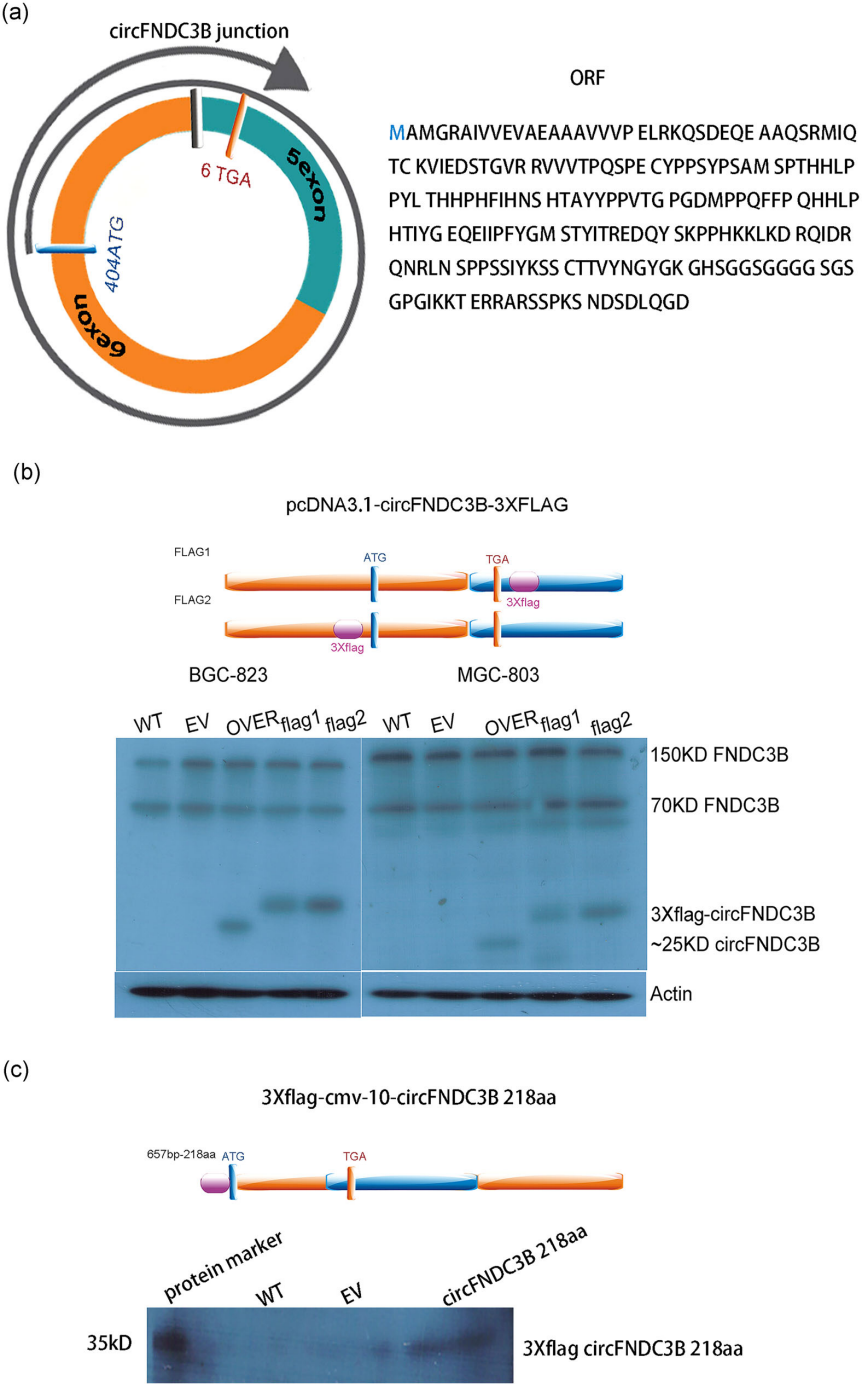
CircFNDC3B has translational protein activity. (a) The figure shows start codon and stop codon positions, 2r+8 rolling circle translation format, and protein sequence information (blue ring: 5 exon; orange ring: 6 exon; black square: junction; blue square: start codon; orange square: stop codon). (b) The circFNDC3B translational protein was detected by inserting a 3× flag–tag at both ends of the initiation codon and the stop codon of the circFNDC3B cyclization vector pcDNA3.1 circFNDC3B mini vector. MGC‐803 cells were transfected with circFNDC3B vector (OVER) or empty vector (EV) or vector with flag–tag for 48 hr. BGC‐823 cells were transfected with circFNDC3B vector (OVER) or empty vector (EV) or vector with flag–tag for 48 hr. Western blot results showed that circFNDC3B could translate approximately 25 kD peptide against the FNDC3B‐specific antibody and could also detect the 150 and 70 kD FNDC3B variants (purple square: 3× flag–tag; blue ring: 5 exon; orange ring: 6 exon; black square: junction; blue square: start codon; orange square: stop codon). (c) A linear expression vector was synthesized and constructed according to the circFNDC3B rolling circle translation sequence. MGC‐803 cells were transfected with p3× flag‐CMV‐10‐circFNDC3B or p3× flag‐CMV‐10 empty vector (EV) for 48 hr. Western blot results showed that circFNDC3B has translational protein activity against flag–tag antibodies (purple square: 3× flag; blue ring: 5 exon; orange ring: 6 exon; black square: junction; blue square: start codon; orange square: stop codon). FNDC3B: fibronectin type III domain‐containing protein 3B [Color figure can be viewed at wileyonlinelibrary.com]

Furthermore, we also predicted that circFNDC3B could translate protein. In the circRNA database (circRNADb, http://reprod.njmu.edu.cn/circrnadb), circFNDC3B had a potential internal ribosome entry site (IRES) and an open reading frame (ORF) of 218aa. The total length of the circFNDC3B rolling circle translation was 657 bp with two sites of ribosome entry sites, 318 to 465 bp and 448 to 524 bp. The start codon was located at 404 bp. The translation from the start codon initiated the first circle translation, and then it entered the 8th bp position after the second circle and terminated at the stop codon (after the junction). Therefore, the ORF had a full length of 2r+8 (Figure 7a). Next, we synthesized two circFNDC3B vectors with 3× flag–tags and transfected the two vectors into cells. The results showed that circFNDC3B could translate an approximately 25 kD peptide (Figure 7b). Moreover, a linear expression vector was synthesized and constructed according to the circFNDC3B rolling circle translation sequence. The results showed that circFNDC3B had translational protein activity against the flag‐ tag antibody (Figure 7c).

## 4 DISCUSSION

In this study, we found that circFNDC3B reduced E‐cadherin expression and enhanced CD44 expression, consequently promoting cell migration and invasion in GC. From this perspective, when circFNDC3B was abnormally expressed, it was correlated with the degree of malignancy and highlighted the cell migration and invasion properties. Ectopic expression of circFNDC3B might serve as a marker of distant metastasis of GC during development and a marker of malignant differentiated cells.

We also overexpressed and silenced circFNDC3B in MGC‐803 cells and BGC‐823 cells, respectively, and then tested the cell cycle and found that neither overexpression nor silencing of circFNDC3B affected cell cycle progression (Figure S2). However, Cell Counting Kit‐8 results showed that the overexpression of circFNDC3B inhibited cell proliferation, whereas silencing of circFNDC3B promoted cell proliferation (Figure S2). From these data, circFNDC3B might play a role in exhibiting migration characteristics rather than cell proliferation in GC. This phenomenon was also observed during embryonic development and partial (intermediate) transitions in cancer cells that allowed the migratory potential of cells while retaining some degree of cell‐cell adhesion, rather than cell proliferation (Theveneau & Mayor, 2011).

In addition, CD44, downstream of circFNDC3B, has complex regulatory mechanisms in cancer development. CD44, the cell surface transmembrane molecule, acting as a cell adhesion molecule, mediates cell motility and responds to the cellular microenvironment (Ponta, Sherman, & Herrlich, 2003; Zoller, 2011). CD44‐hyaluronan interactions have been associated with cancer invasion in the EMT (Toole, 2009; Warzecha & Carstens, 2012). Furthermore, CD44 expression can lead to high malignancy and poor prognosis and can consequently resist therapy (Anido et al., 2010; Visvader & Lindeman, 2008; Yoon et al., 2014). On the one hand, CD44 is a marker of cancer stem cell (CSC) mediated tumorigenesis (Polyak & Weinberg, 2009) and triggers the EMT (Bhattacharya, Mitra, Chaudhuri, & Roy, 2017; Ghuwalewala et al., 2016). These processes are both important outcomes affecting invasiveness and metastasis (Sleeman & Cremers, 2007; Thiery & Sleeman, 2006). The relationship between the EMT and CSCs is controversial (Nieto, 2013), although the EMT can confer stem cell‐like properties in cells (Celia‐Terrassa et al., 2012; Nieto et al., 2016; Polyak & Weinberg, 2009). In addition, malignant cancer requires the reversion to epithelial phenotype and maintenance of the “stemness” state (van Denderen & Thompson, 2013). This is in accordance with our data showing that BGC‐823 also presented an epithelial phenotype with high expression of E‐cadherin. Our results suggested that overexpressing circFNDC3B only reduced E‐cadherin levels, whereas silencing circFNDC3B not only increased E‐cadherin levels but also reduced N‐cadherin, SNAI1, and Vimentin levels (Figure 4). It was consistent with the phenotypic results that promoted cell migration, but why did overexpression only affect the level of E‐cadherin that presented epithelial traits? Was it because of the partial (intermediate) transitions in EMT? Therefore, there are always a number of studies based on this CSC concept, and the correction of EMT is being investigated (Batlle & Clevers, 2017; Ramos, Hoffmann, Gerson, & Liu, 2017). On the other hand, CD44 is a controversial molecule that has been shown to have both positive and negative correlations with survival at the nonmonotonic level by controlling cell migration and invasion (Klank et al., 2017). Thus, these controversial discoveries suggest that the simple inhibition of CD44 in clinical therapy is not effective and may result in a hypermigratory state. From the different expression of circFNDC3B in GC cells and the results of inhibition of cell proliferation, whether circFNDC3B also has the ability to control migration at the nonmonotonic level is unknown.

Interestingly, primary *FNDC3B* mRNA transcripts generated circFNDC3B via back‐splicing and circularization of selected exons, which further increased the complexity of genome output to contend FNDC3B linear mRNA. According to our data, circFNDC3B contributed to modulation of the counterpart, FNDC3B protein, which is associated with cell migration (Cai et al., 2012), deriving from the linear mRNA. Furthermore, analysis of the circFNDC3B sequence revealed the presence of an IRES and ORF. The coding ability of circFNDC3B was proven through the use of artificial constructs with 3× flag–tags expressing circular transcripts. Our data demonstrated that circFNDC3B could translate an approximately 25 kD peptide (Figure 7). By using the Simple Modular Architecture Research Tool (http://smart.embl.de; Letunic & Bork, 2018), we found that the circular transcripts had multiple low complexity regions; however, FNDC3B had multiple FN3 domains which played a role in cell adhesion, cell morphology, thrombosis, cell migration, and embryonic differentiation (Leahy, Aukhil, & Erickson, 1996; Leahy, Hendrickson, Aukhil, & Erickson, 1992). Therefore, we still had no hints on the molecular activity of the peptides derived from circFNDC3B or whether they play a control role in cell migration or other roles.

Overall, despite our studies showing that circFNDC3B affects E‐cadherin and CD44 levels, several questions still need to be further clarified. First, we detected the relationship between circFNDC3B, IGF2BP3, and *CD44* mRNA in pull‐down components, but we do not yet know there were other unknown binding molecules. Second, the molecular mechanism and correlation between circFNDC3B and *FNDC3B* linear mRNA are unclear. Third, we investigated whether the peptides derived from circFNDC3B play a role in GC. All these questions need further investigation and will orient our continued exploration of the role of circFNDC3B in GC progress.

## CONFLICT OF INTERESTS

The authors declare that they have no conflict of interests.

## AUTHOR CONTRIBUTIONS

H. Y., Q. H., and L. Y. participated in all experimental work and drafted the paper. H. Y. participated in the design of the experimental protocols. Z. Y., Z. X., and L. L. were involved in the part of experiments. L.K., L. L., and D. X. performed data analysis. L. F., S. S., and L. G. designed the experimental protocols.

## Supporting information

Supporting information
