# Supplementary material for: FNDC3B circular RNA promotes the migration and invasion of gastric cancer cells via the regulation of E‐cadherin and CD44 expression
Source: J Cell Physiol. 2019 Apr 8;234(11):19895–910. doi: 10.1002/jcp.28588 (PMC6766960; doi:10.1002/jcp.28588)
Supplement: Supplementary file 1 — Supporting information [file JCP-234-19895-s001.doc]

**
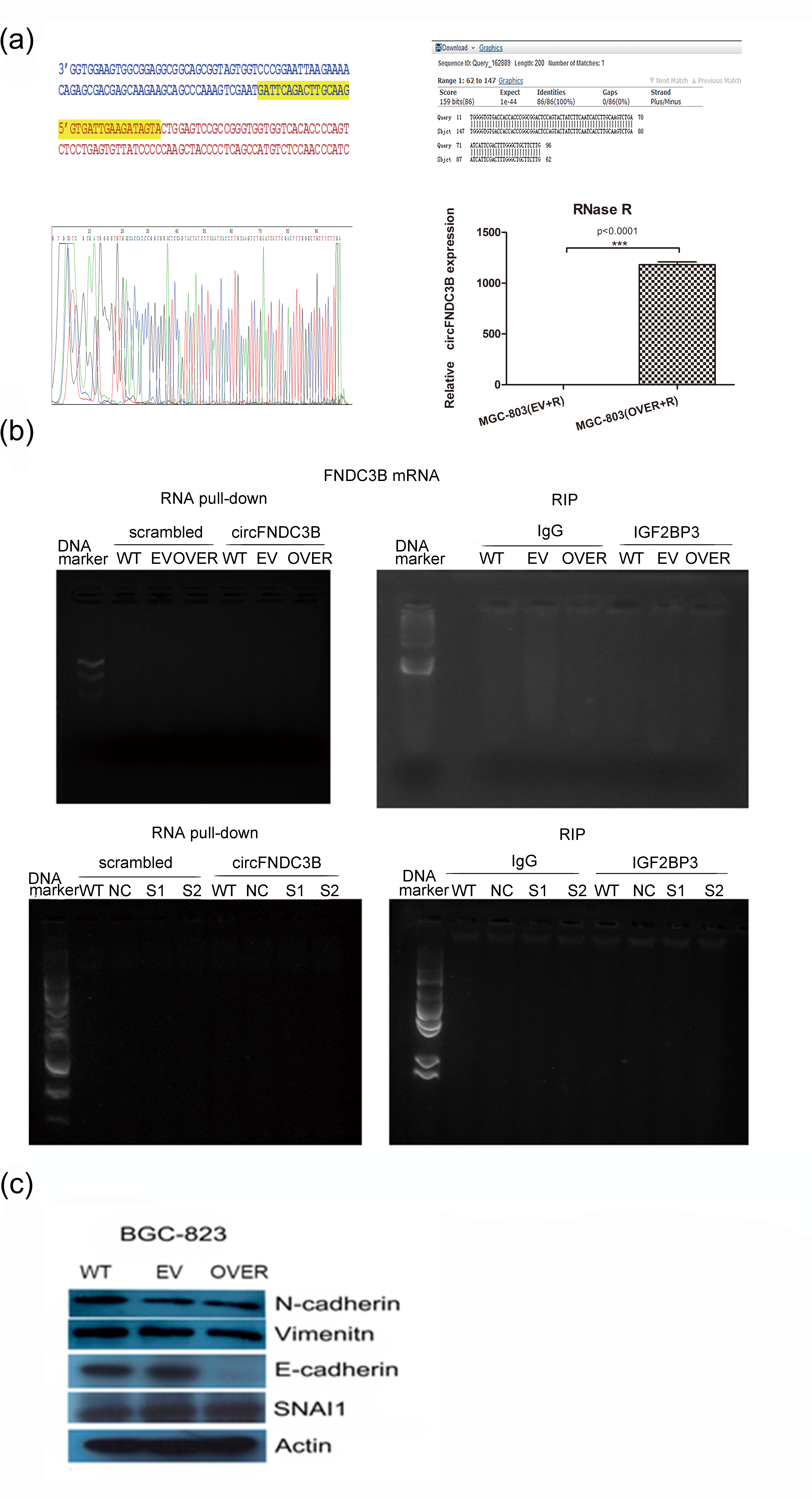
**

**Supporting Information Figure 1.** The sequence of circFNDC3B and its junction sequence, and *FNDC3B* mRNA did not interact with IGF2BP3 and circFNDC3B. (a) circFNDC3B was circularized by exons 5 and 6 of *FNDC3B* pre-mRNA, and Sanger sequencing showed the back-spliced events of circFNDC3B. BLAST alignment showed a highly complementary circFNDC3B junction sequence circularized by the vector. qRT-PCR showed resistance of circFNDC3B to RNase R digestion in overexpression of circFNDC3B in MGC-803. (b) MGC-803 cells were transfected with circFNDC3B vector (OVER) or empty vector (EV) for 48 hr. BGC-823 cells were transfected with siRNA (S1, S2) or negative control siRNA (NC) for 48 hr. RIP assays and RNA pull-down assays were performed to assess the interaction of *FNDC3B* mRNA, circFNDC3B and IGF2BP3. MGC-803 cells were transfected with circFNDC3B vector (OVER) or empty vector (EV) for 48 hr. (c) The results showed that circFNDC3B affected the marker proteins of EMT in BGC-823 cells transfected with the circFNDC3B vector. Data were expressed as the mean ± SEM and were analyzed by independent sample *t*-test, n=3.*p<0.05, **p<0.01, ***p<0.0001.

**
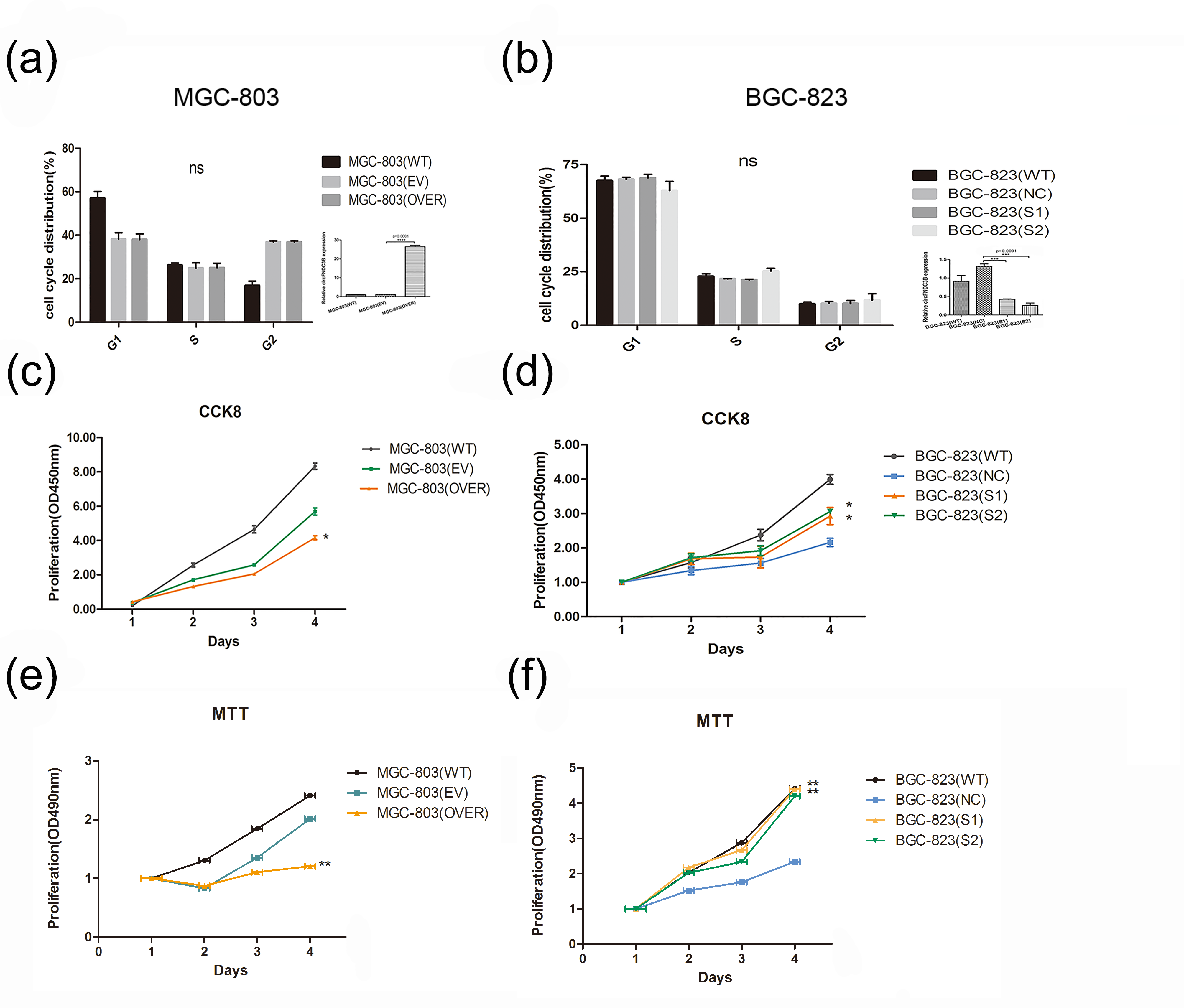
**

**Supporting Information Figure 2.** CircFNDC3B inhibited cell proliferation. (a, b) We overexpressed and silenced circFNDC3B in MGC-803 cells and BGC-823 cells, respectively, and then tested the cell cycle. The results suggested that neither overexpression nor silencing of circFNDC3B affected cell cycle progression. The bottom right corner showed that successful overexpression of circFNDC3B and silencing of circFNDC3B (WT: wild-type, EV: empty vector, OVER: overexpressed circFNDC3B, NC: negative control, siRNA:S1, S2). (c, d) The CCK-8 assay showed that overexpression of circFNDC3B inhibited cell proliferation, while silencing circFNDC3B promoted cell proliferation (WT: wild-type, EV: empty vector, OVER: overexpressed circFNDC3B, NC: negative control, siRNA: S1, S2). (e, f) Similarly, an MTT assay was performed to detect cell proliferation, and the results suggested that circFNDC3B inhibited cell proliferation. (WT: wild-type, EV: empty vector, OVER: overexpressed circFNDC3B). Data were expressed as the mean ± SEM and were analyzed by independent sample *t*-test, n=3.*p<0.05, **p<0.01, ***p<0.0001.

Supporting Information Table 1. Primers for qRT-PCR and vector.

| GAPDH-F | ACCACAGTCCATGCCATCAC |
| --- | --- |
| GAPDH-F | TCCACCACCCTGTTGCTGTA |
| Actin-F | CATGTACGTTGCTATCCAGGC |
| Actin-R | CTCCTTAATGTCACGCACGAT |
| 18s-F | TTAATTCCGATAACGAACGAGA |
| 18s-R | CGCTGAGCCAGTCAGTGTAG |
| U6-F | CTCGCTTCGGCAGCACA |
| U6-R | AACGCTTCACGAATTTGCGT |
| FNDC3B mRNA-F | CAGAGCCCCGCGTTTCA |
| FNDC3B mRNA-R | GGCATCATGGCTACCTCTCC |
| circ-FNDC3B-F | CAAGAAGCAGCCCAAAGTCG |
| circ-FNDC3B-R | R-CATGGCTGAGGGGTAGCTTG |
| IGF2BP3-F | ACTGCACGGGAAACCCATAG |
| IGF2BP3-R | CCAGCACCTCCCACTGTAAAT |
| CD44-F | CATCAGTCACAGACCTGCCCAATGC |
| CD44-R | ATGTAACCTCCTGAAGTGCTGCTCC |
| S1-IGF2BP3 | GUUGUAAAUGUAACCUAUUdTdT  AAUAGGUUACAUUUACAACdTdT |
| S2-IGF2BP3 | CAUAAGGAAGCUCAAGAUUAdTdT  UAUCUUGAGCUUCCUUAUGdTdT |
| S3-IGF2BP3 | CAGGAAUUGACGCUGUAUAdTdT  UAUACAGCGUCAAUUCCUGdTdT |
| Negative Control | UUCUCCGAACGUGUCACGUdTdT  ACGUGACACGUUCGGAGAAdTdT |
